# Enhanced Pulmonary Vascular and Alveolar Development via Prenatal Administration of a Slow-Release Synthetic Prostacyclin Agonist in Rat Fetal Lung Hypoplasia

**DOI:** 10.1371/journal.pone.0161334

**Published:** 2016-08-16

**Authors:** Satoshi Umeda, Shigeru Miyagawa, Satsuki Fukushima, Noriko Oda, Atsuhiro Saito, Yoshiki Sakai, Yoshiki Sawa, Hiroomi Okuyama

**Affiliations:** 1 Department of Pediatric Surgery, Osaka University Graduate School of Medicine, Osaka, Japan; 2 Department of Cardiovascular Surgery, Osaka University Graduate School of Medicine, Osaka, Japan; MEXICO

## Abstract

Lung hypoplasia and pulmonary hypertension are the major causes of mortality in neonates with congenital diaphragmatic hernia (CDH). Although the prostaglandin pathway plays a pivotal role in lung development, the reported efficacy of postnatal prostaglandin agonist treatment is suboptimal. We hypothesized that prenatal treatment with ONO-1301SR, a slow-release form of a novel synthetic prostacyclin agonist with thromboxane inhibitory activity, might enhance the development of lungs exhibiting hypoplasia in the fetal period. On embryonic day (E) 9.5, nitrofen was given to pregnant Sprague-Dawley rats to establish a CDH-related lung hypoplasia model, whereas normal rats received the vehicle only. The same day, either ONO-1301SR or a placebo was also randomly administered. On E21.5, the fetuses of the normal group and those exhibiting CDH were analyzed. Prenatal ONO-1301SR administration had no influence on the incidence of nitrofen-induced CDH. The lung-to-body weight ratio in the CDH+ONO group was greater than that in the CDH group. Histologically, the medial wall in the CDH+ONO group was two-thirds thinner than that in the CDH group. In addition, the number of Ttf-1-positive cells and the capillary density were ≥1.5 times greater in the CDH+ONO group than in the CDH group, and this increase was associated with higher expression of vascular endothelial growth factor and stromal cell-derived factor in the CDH+ONO group, suggesting enhanced development of the alveolar and capillary networks. Thus, prenatal ONO-1301SR was protective against the progression of lung hypoplasia associated with CDH in a nitrofen-induced rat model, indicating the potential of this treatment for pathologies exhibiting lung hypoplasia.

## Introduction

Lung hypoplasia and pulmonary hypertension are the major causes of mortality in neonates with congenital diaphragmatic hernia (CDH). Although the etiology of lung hypoplasia or pulmonary hypertension varies, lung development in the fetal period is associated with the prostaglandin pathway, wherein prostacyclin regulates fetal pulmonary vascular tone and lung vessel development. Postnatal administration of prostaglandin analogues such as epoprostenol might therefore be considered as a promising treatment strategy; however, the efficacy of this treatment is reportedly limited, at least in part because of the timing of the treatment or development of drug resistance, which is known to be substantial for commercially available prostaglandin analogues.

The nitrofen-induced CDH rat model is a well-established experimental model, which recapitulates the pulmonary abnormalities observed in human CDH, including lung hypoplasia and pulmonary vascular remodeling. Some possible prenatal treatments were previously tested using this model, which were found to decrease the pulmonary vasculature, including steroids [1], sildenafil citrate [2], and retinoic acid [3]. However, these previous reports for treating fetal lung hypoplasia required frequently repeated administration of the drugs, and clinical trials have not yet been realized. ONO-1301 is a synthetic prostacyclin receptor agonist that is chemically and biologically stable because of the absence of typical prostanoid structures, and importantly has thromboxane A2 inhibitory activity that minimizes drug resistance [4]. Of note, repeated administration of ONO-1301 attenuated pulmonary vascular remodeling and improved survival in a monocrotaline-induced pulmonary hypertension rat model [5], in association with upregulation of endogenous vascular endothelial growth factor (VEGF), hepatocyte growth factor (HGF), and stromal cell-derived factor (SDF)-1 [6]. Furthermore, we have developed a polylactic-co-glycolic acid copolymer-polymerized microsphere form of ONO-1301 (ONO-1301SR), which provided a sustained ONO-1301 effect and attenuated monocrotaline-induced pulmonary hypertension in rats with a single injection [7].

We therefore hypothesized that prenatal maternal administration of ONO-1301SR might enhance the development of the hypoplastic lung in fetuses with CDH. To test this hypothesis, we used a well-established experimental nitrofen-induced CDH rat model that recapitulates the pulmonary abnormalities observed in human CDH, including lung hypoplasia and pulmonary vascular remodeling [8].

## Methods

### Ethical considerations

All procedures and protocols were approved by the Committee on the Ethics of Animal Experiments of the Osaka University Graduate School of Medicine, Suita, Osaka, Japan.

### Animal model

Pregnant Sprague-Dawley rats (Oriental Yeast Co., Osaka, Japan) were gavage-fed with 100 mg of the herbicide nitrofen (2,4-dichloro-4-nitrofenil ether; Tokyo Chemical Industry, Tokyo, Japan) dissolved in 1.0 mL of olive oil at embryonic day (E) 9.5 (predicted term: E22, n = 12) as previously described [9], whereas control rats received olive oil only (n = 4). The dose and administration timing of ONO-1301SR were determined based on the results of a preliminary study. Of the 12 nitrofen-administered rats, 5 received a single subcutaneous injection of 30 mg/kg ONO-1301SR (Ono Pharmaceutical Co. Ltd., Osaka, Japan) dissolved in saline at E9.5. The following 3 groups were thus generated: control (n = 4), nitrofen administration (n = 7), and nitrofen+ONO-1301SR administration (n = 5). At term (E21.5), the pregnant rats were anesthetized and the fetuses were harvested by cesarean section. Fetuses were weighed and examined for diaphragmatic defects, and the lungs were harvested for further pathobiological evaluation. The fetuses that had macroscopically developed diaphragmatic defects in the nitrofen and nitrofen+ONO-1301SR groups were diagnosed with CDH and were further analyzed.

### Assay of ONO-1301 plasma levels

Maternal and fetal (E21.5) blood was sampled, the fetal blood samples (median n = 15, range: 13–15 fetuses per dam) were pooled (200 μL), and plasma was isolated from these samples by centrifugation at 3,000 rpm for 15 min. Plasma ONO-1301 levels were measured using liquid chromatography tandem mass spectrometry.

### Total DNA content in the fetal lung

Snap-frozen lungs (n = 5 fetuses per group) were homogenized, and the total DNA of each lung was extracted using a commercially available kit (Genomic-tip 100/G and Genomic DNA Buffer Set; Qiagen, Hilden, Germany). The total DNA content was measured using a spectrophotometer (Multiskan GO; Thermo Scientific, Rockford, IL, USA).

### Lung morphometry

Fetal rat lungs from each group were fixed by tracheal instillation of 10% buffered formalin under a constant pressure of 20 cm H_2_O. After ligation of the trachea, the lungs were immersed in fixative, embedded in paraffin, and sectioned into 4-μm serial coronal sections for further morphometric and immunohistochemical analyses. Sections stained with hematoxylin and eosin (H&E) were used for pulmonary airspace assessment by measuring the mean linear intercept (Lm) as previously described [10]. In each sample, 10 fields were measured and their values averaged (n = 6 fetuses per group). Sections in which elastin was stained with van Gieson’s stain were used for pulmonary artery (PA) remodeling assessment. The PAs were distinguished from pulmonary veins based on their position and structure. To assess PA remodeling, the percentage of the medial wall thickness was calculated according to the following formula: (2 × medial wall thickness/external diameter) × 100% [11]. In each sample, only small PAs (external diameter: 30 to 100 μm) that were approximately round (i.e., maximal diameter of the external diameter did not exceed the minimal diameter by >50%) were measured and their values were averaged (n = 6 fetuses per group) [12]. Morphometric assessments were performed using a Biorevo BZ-9000 system (Keyence, Osaka, Japan) with 400× magnification.

### Immunohistochemistry

The 4-μm serial coronal sections of the fetal rat lungs were deparaffinized and washed. Epitope retrieval was performed using Target Retrieval Solution (Dako, Glostrup, Denmark). The sections were then washed with phosphate buffered saline and nonspecific binding was blocked with Protein Block, Serum Free (Dako). Primary antibodies targeting the following proteins were used for immunohistochemistry: α-smooth muscle actin (SMA, 1:50, mouse, Dako), prostacyclin receptor (1:150, mouse, Abcam, Cambridge, UK), Ki-67 (1:50, rabbit, Abcam), and Ttf-1 (1:50, mouse, Abcam). The primary antibody staining was visualized using fluorescence-conjugated secondary antibodies (Life Technologies, Grand Island, NY, USA). Isolectin GS-B4 (ILB4, 1:25, Life Technologies) was included during incubation with the secondary antibodies for vascular endothelial cell staining. Hoechst33342 was used after incubation with the secondary antibodies for nuclear counterstaining. The Ki-67-positive cell number was quantified by counting the Ki-67-positive and negative nuclei within the medial layers of the PA that were positive for α-SMA. The ILB4-positive vascular area was quantified by measuring the positive area under 400× magnification. These assessments were performed using a Biorevo BZ-9000 system (Keyence) under 400× magnification.

### Quantitative real-time polymerase chain reaction (PCR)

Total RNA was extracted from the lungs (n = 5 fetuses per group) using the RNeasy Kit and reverse-transcribed using the SuperScript VILO Master Mix (Life Technologies). Real-time PCR was performed using the 7500 Fast Real-Time PCR System (Applied Biosystems, Foster City, CA, USA) using primers for *Vegf* (Rn01511601_m1), *Hgf* (Rn00566673_m1), *Sdf1* (Rn00573260_m1), and *Gapdh* (Rn01775763_g1) (Life Technologies). The average copy numbers of the gene transcripts were normalized to those of the *Gapdh* gene.

### Total protein content and western blot analysis in the fetal lung

Snap-frozen lungs (n = 4 fetuses per group) were homogenized on ice in homogenization buffer (RIPA buffer; Thermo Scientific) containing a protease inhibitor cocktail tablet (Roche Diagnostics GmbH, Mannheim, Germany). Homogenized samples were sonicated and centrifuged at 10,000 *g* for 20 min at 4°C. The total protein content in the lung was quantified using a BCA protein assay kit (Thermo Scientific). The total proteins (30 μg per lane) were subjected to sodium dodecyl sulfate-polyacrylamide gel electrophoresis, and proteins from the gel were then transferred to polyvinylidene fluoride membranes by electroblotting. Immunodetection was performed using a rabbit anti-VEGF polyclonal antibody (Abcam), diluted at 1:1,000, overnight at 4°C. After the blots were washed to remove unbound antibody, an anti-rabbit horseradish peroxidase antibody (Cell Signaling, Danvers, MA, USA), diluted at 1:10,000, was applied as the secondary antibody for 1 h at room temperature. After the membrane was washed, the bands were visualized by enhanced chemiluminescence using ImmunoStar LD (Wako, Osaka, Japan). In addition, each gel was stripped and reprobed with GAPDH to normalize for protein loading.

### Statistical analysis

Values are expressed as the mean ± SEM. Statistical analyses were performed using JMP software (SAS Institute Inc., Cary, NC, USA). Normality of the data distribution was confirmed by the Bartlett’s test. Comparisons of the parameters between 2 groups were performed using one-way ANOVA followed by Tukey’s honest significant difference post-hoc test. Statistical significance was defined as *P* < 0.05.

## Results

### Rationale underlying maternal administration of ONO-1301SR for treating the fetal lung

**Fig 1:**
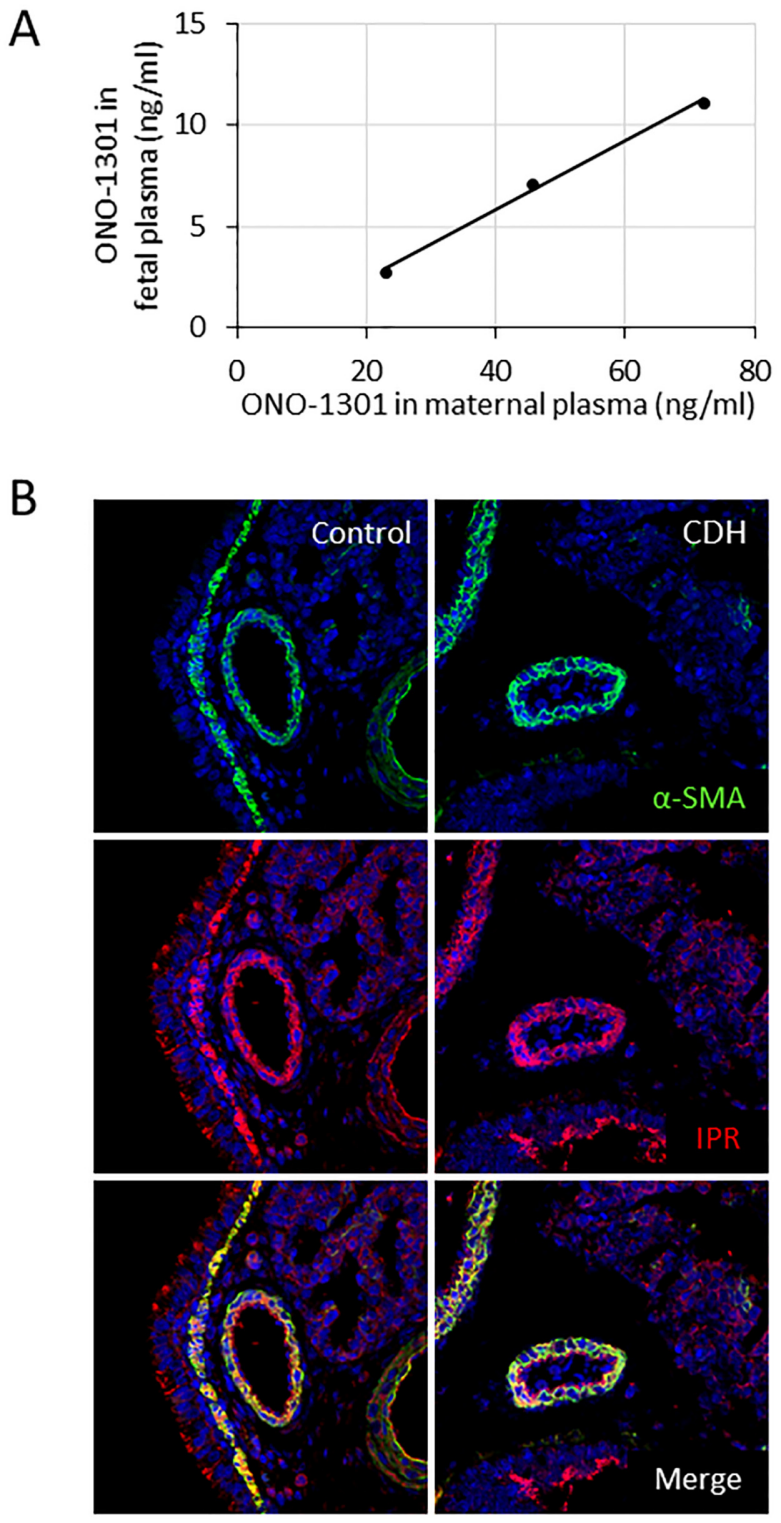
Correlation of the maternal and fetal plasma ONO-1301 concentrations, and the location of prostacyclin receptor (IPR) expression in control fetal lungs and those with congenital diaphragmatic hernia (CDH). One-seventh of the maternal blood concentration of ONO-1301 was transferred to the fetal blood at E21.5, demonstrating the efficient placental permeability of this molecule (**A**). Immunohistochemistry images showing coexpression of IPR in α-smooth muscle actin (α-SMA)-positive cells in both control and CDH fetal lungs (**B**).

The placental permeability of ONO-1301 was investigated by comparing maternal and fetal plasma ONO-1301 concentrations. The maternal and fetal plasma concentrations of ONO-1301 were significantly correlated (Fig 1A). The fetal concentration was approximately one-seventh of the maternal concentration, suggesting that ONO-1301 was permeable across the placenta.

In addition, the distribution of the prostacyclin receptor (IPR), the specific ligand of ONO-1301, in the fetal lung was investigated by immunohistochemical labeling. The control and the CDH groups displayed similar IPR distributions in the fetal lungs (Fig 1B). IPR was predominantly expressed in the α-SMA-positive cells constituting the pulmonary artery, in contrast to the endothelial cells or fibroblasts that showed only slight IPR expression.

### Effects of prenatal ONO-1301 on macroscopic development of the fetal lungs

**Fig 2:**
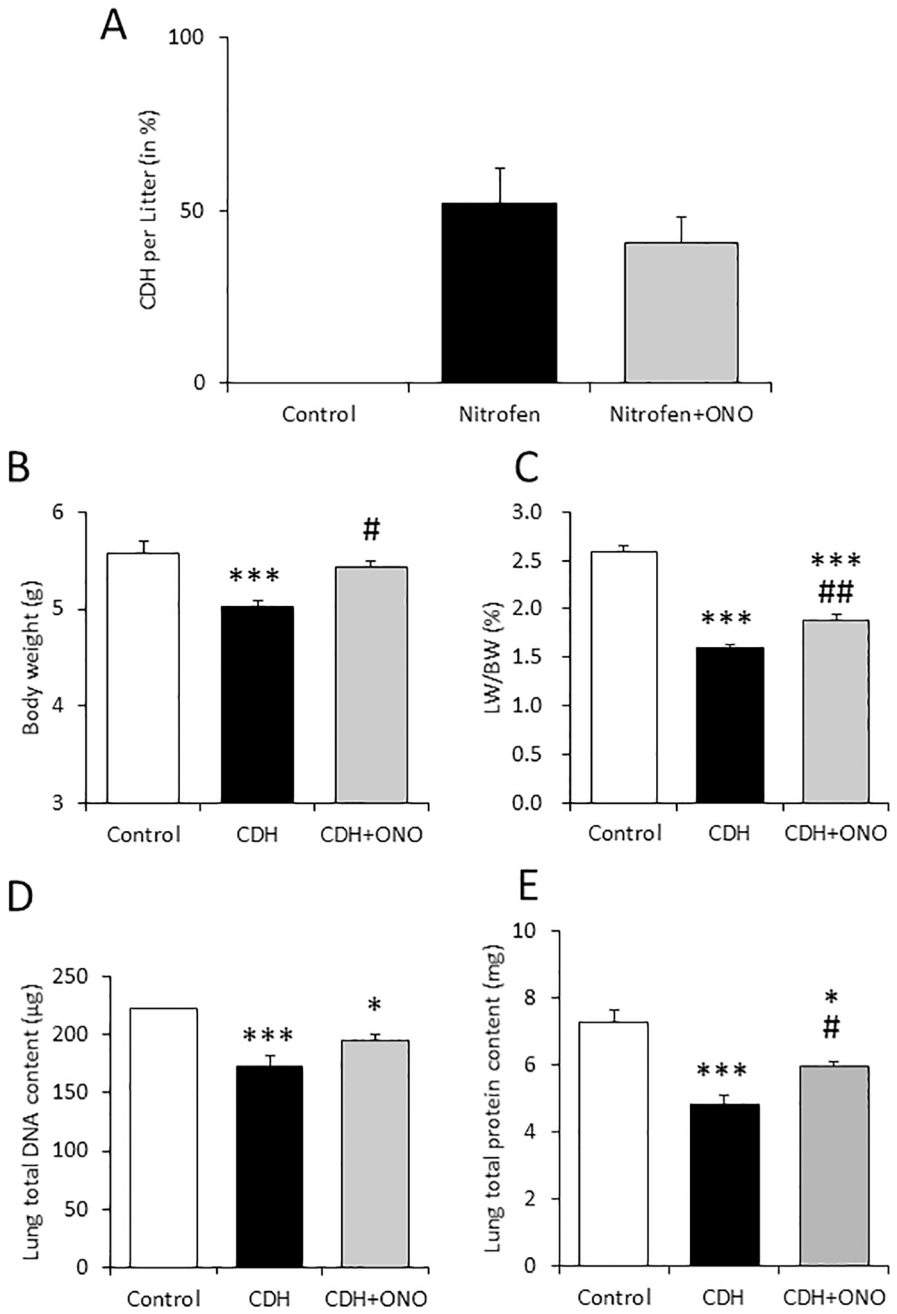
Effects of prenatal ONO-1301SR treatment on the macroscopic development of fetal hypoplastic lungs. The incidence of congenital diaphragmatic hernia (CDH) (**A**), fetal body weight (**B**), lung-to-body weight ratio (**C**), total DNA (**D**), and total protein content (**E**) in the fetal lung were evaluated in each pup of the control (white bars), nitrofen-induced CDH (black bars), and nitrofen-induced CDH treated with ONO-1301SR (CDH+ONO; gray bars) groups. The values are expressed as the mean ± SEM. * 0.01 < *P* < 0.05, *** *P* <0.001 versus control fetuses; # 0.01 < *P* < 0.05, ## 0.001< *P* < 0.01 versus nitrofen-CDH fetuses.

Nitrofen was orally administered, with or without subcutaneous injection of ONO-1301SR, to pregnant rats at E9.5 to induce CDH-associated lung hypoplasia. Approximately 50% of the nitrofen-treated fetuses developed macroscopic CDH, and prenatal ONO-1301SR treatment did not influence the incidence of the diaphragmatic defect (Fig 2A). ONO-1301SR treatment also did not affect the macroscopic development of the other organs.

The nitrofen-induced CDH fetuses had a significantly smaller body weight at E21.5 than the control fetuses to which nitrofen was not given, whereas the fetuses prenatally treated by ONO-1301SR displayed a significantly greater body weight than the fetuses that did not receive the treatment (Fig 2B). The lung-to-body weight ratio (LW/BW), an indicator of lung hypoplasia, was significantly lower in the CDH fetuses without the ONO-1301SR treatment than in the controls. In contrast, the fetuses receiving ONO-1301SR treatment displayed a significantly higher LW/BW than those without treatment, and a significantly lower LW/BW than the control fetuses (Fig 2C). The total DNA and protein contents in the fetal lung, indicators of organ cell mass, were significantly smaller in the fetuses with CDH, regardless of ONO-1301SR treatment, compared to the controls. Although the total DNA content in the ONO-1301SR-treated fetal CDH lungs was not significantly different from that in the fetal CDH lungs without ONO-1301SR, the total protein content in the former group was significantly higher (Fig 2D and 2E).

### Preservation of lung structure in CDH fetuses by prenatal ONO-1301SR treatment

**Fig 3:**
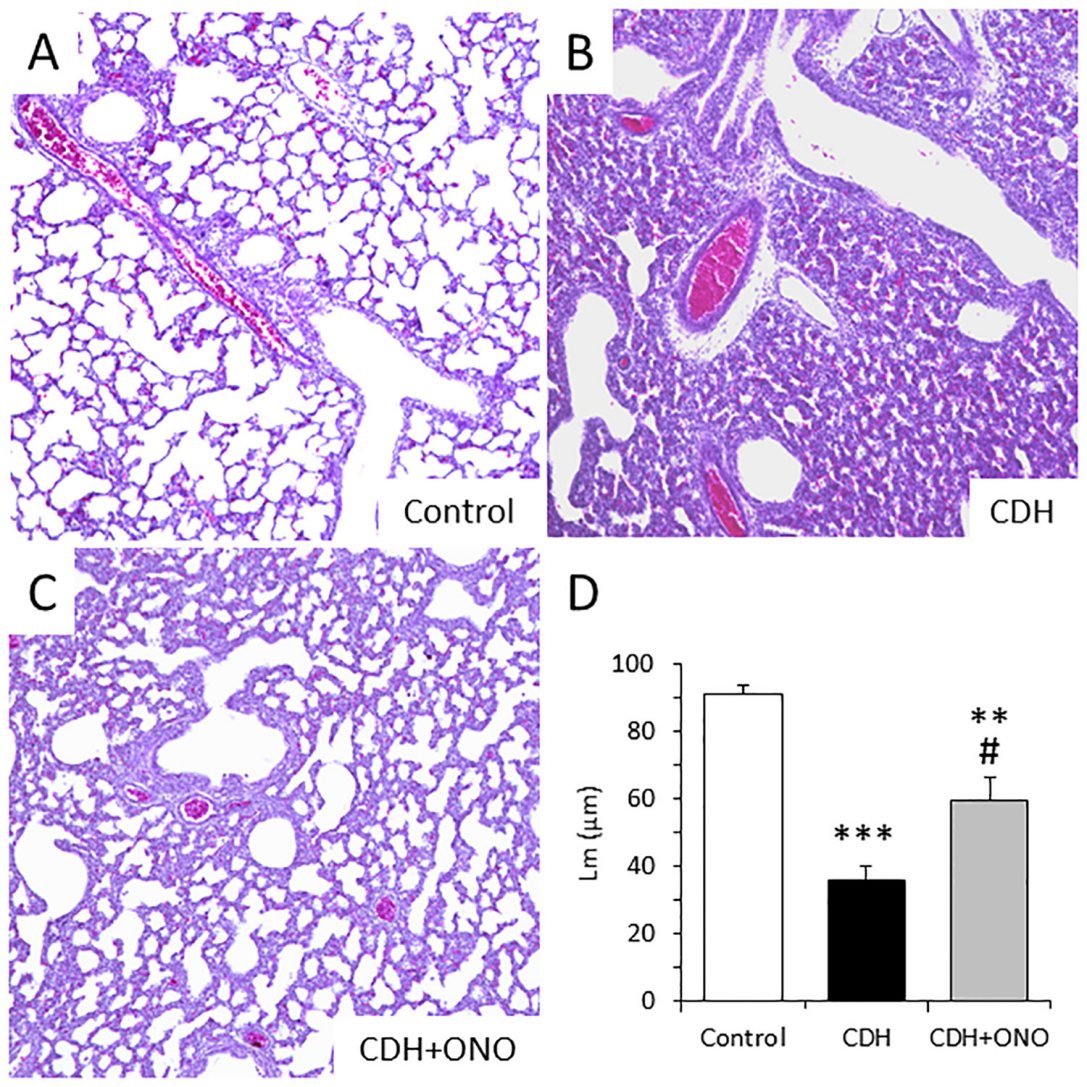
Preservation of lung structure in fetuses with congenital diaphragmatic hernia (CDH) following prenatal ONO-1301SR treatment. Representative hematoxylin-eosin stained lung sections from control (**A**), CDH (**B**), and CDH + ONO-1301SR-treated (**C**) fetuses at 100× magnification. The mean linear intercept (Lm, in μm) in the lungs from the control (white bar; n = 6), CDH (black bar; n = 6), and CDH + ONO-1301SR-treated (gray bar; n = 6) groups are shown (**D**). The values are expressed as the mean ± SEM. ** 0.001 < *P* <0.01, *** *P* < 0.001 versus control fetuses; # 0.01 < *P* < 0.05 versus nitrofen-CDH fetuses.

The microscopic structure of the lungs in the CDH fetuses was assessed in comparison to the control fetuses by H&E staining. The nitrofen-induced CDH fetuses displayed significantly smaller pulmonary airspaces and lower mean linear intercept values than the controls (Fig 3A and 3B), whereas CDH fetuses treated with ONO-1301SR displayed preservation of the pulmonary airspace when compared to fetuses without ONO-1301SR treatment (Fig 3C). The mean linear intercept value in the ONO-1301SR-treated fetuses was significantly higher than that in the fetuses without the treatment (Fig 3D).

**Fig 4:**
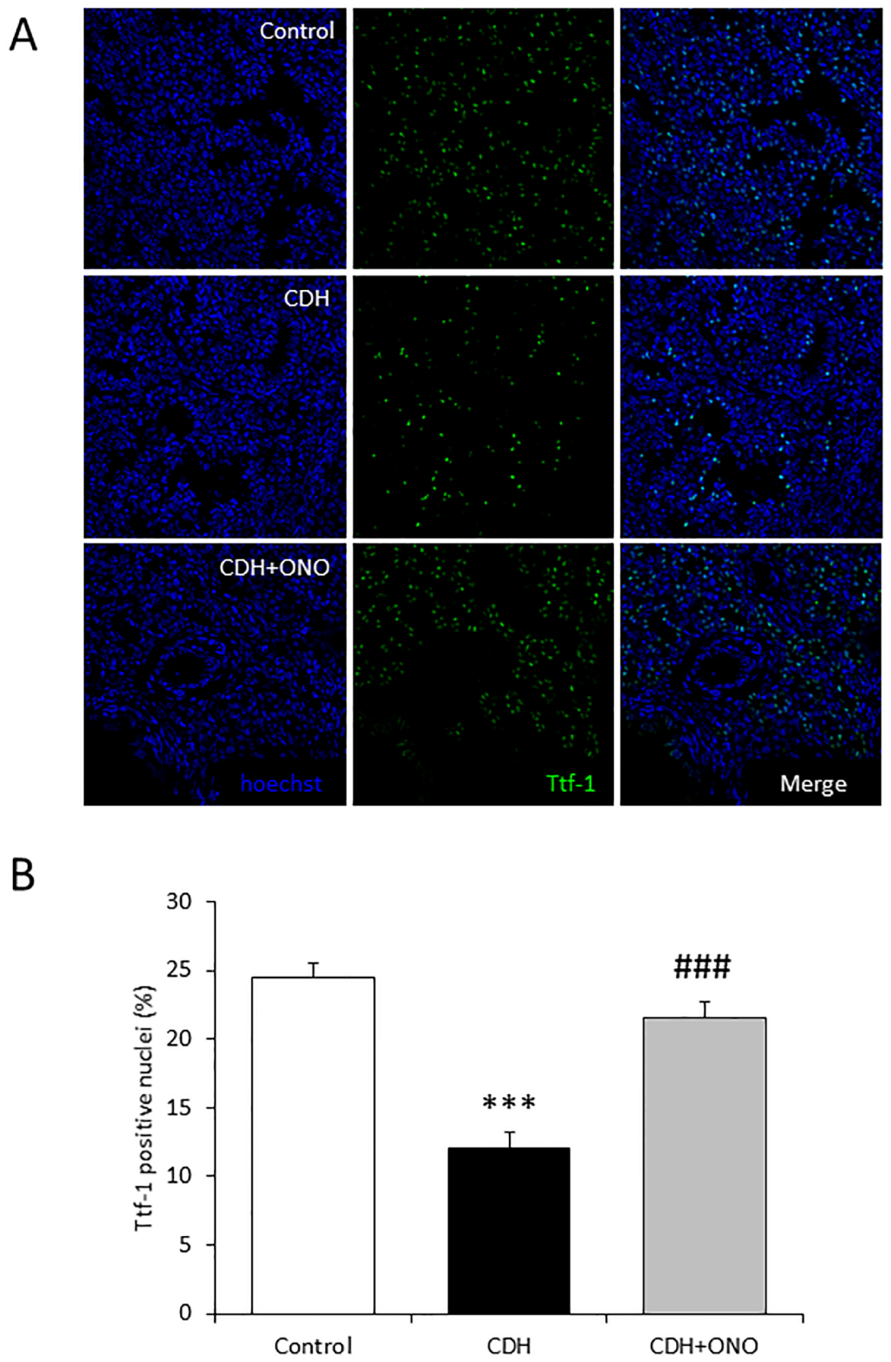
Enhanced alveolar development in fetuses with congenital diaphragmatic hernia (CDH) following prenatal ONO-1301SR treatment. Representative Ttf-1 immunostaining in fetal lung sections from control, CDH, and CDH + ONO-1301SR-treated pups at 400× magnification (**A**). The percentage of Ttf-1 positive cells determined relative to the total number of cells in the lungs from control (white bar; n = 6), CDH (black bar; n = 6), and CDH + ONO-1301SR-treated (gray bar; n = 6) pups (**B**) are shown. The values are expressed as the mean ± SEM. *** *P* < 0.001 versus control fetuses; ### *P* < 0.001 versus nitrofen-CDH fetuses.

In addition, the distribution and quantity of the alveolar epithelium in the CDH fetuses were assessed in comparison to the control fetuses by immunohistolabeling of Ttf-1, a specific marker of type II alveolar epithelial cells. The Ttf-1-positive alveolar epithelium was dispersed in the nitrofen-induced CDH fetal lungs, in contrast to the controls that showed a well-structured Ttf-1-positive alveolar epithelium, whereas the ONO-1301SR-treated CDH lungs displayed preservation of the Ttf-1-positive alveolar epithelium compared to lungs without ONO-1301SR treatment (Fig 4A). Furthermore, the Ttf-1-positive cell number was significantly smaller in the nitrofen-induced CDH fetuses without, but not with, ONO-1301SR treatment compared to the controls (Fig 4B).

**Fig 5:**
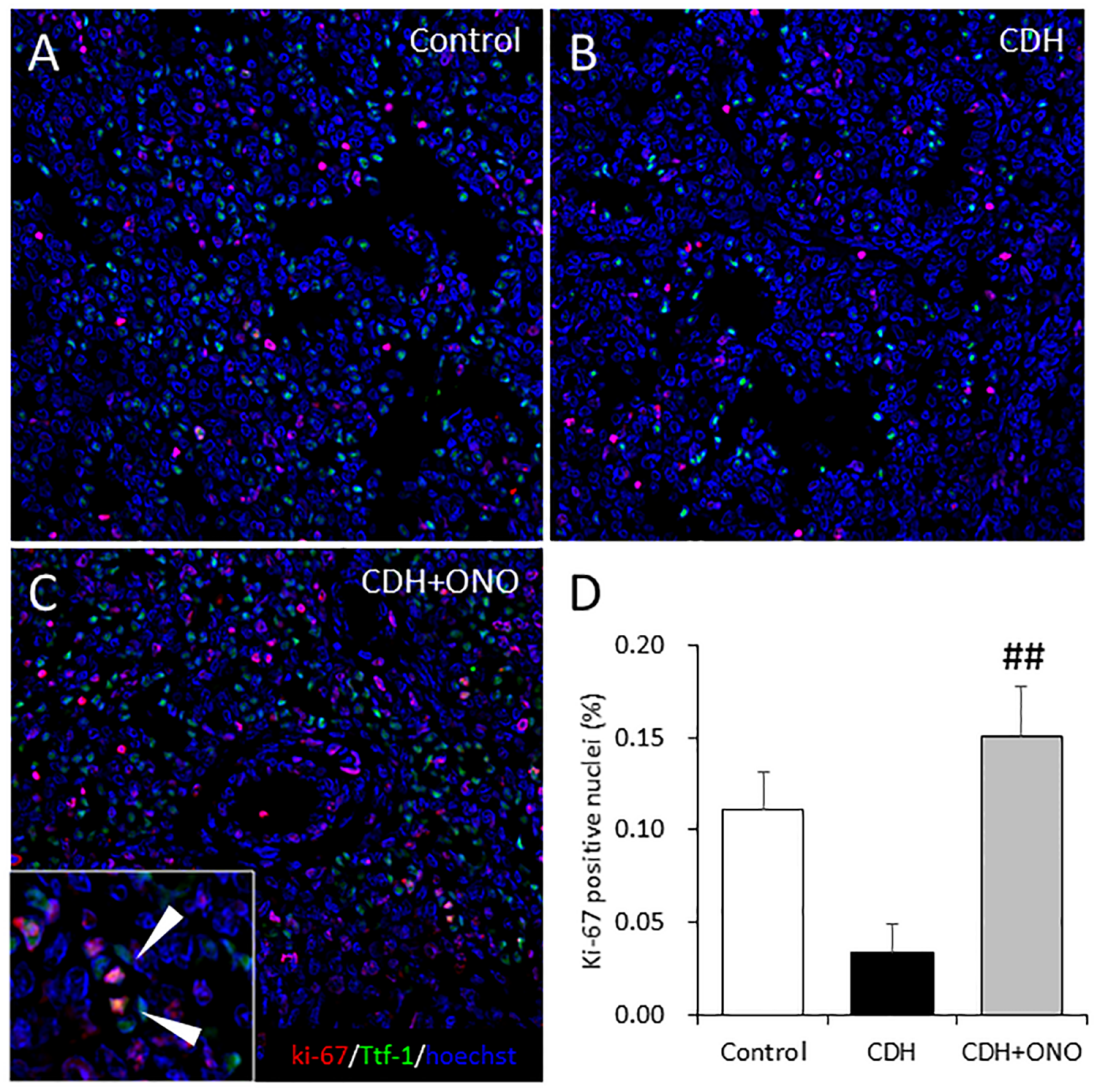
Enhanced alveolar epithelial cell proliferation in fetus with congenital diaphragmatic hernia (CDH) following prenatal ONO-1301SR treatment. Representative Ttf-1 and Ki-67 immunostaining images of fetal lung sections from control (**A**), CDH (**B**), and CDH + ONO-1301SR-treated (**C**) pups at 400× magnification. The percentage of Ttf-1 and Ki-67 double-positive cells (white arrows) relative to the total number of cells in the lungs from control (white bar; n = 6), CDH (black bar; n = 6), and CDH + ONO-1301SR-treated (gray bar; n = 6) pups (**D**) are shown. The values are expressed as the mean ± SEM. ## 0.001 < *P* <0.01, versus nitrofen-CDH fetuses.

In addition, the proliferative activity of the Ttf-1-positive cells in the CDH lungs was compared to that in the control lungs by immunohistolabeling of Ttf-1 and Ki-67. Ttf-1 and Ki-67 double-positive cells were dispersed in the CDH lungs without the ONO-1301SR treatment (Fig 5A–5C) and were significantly fewer in number (Fig 5D) than in the control and CDH lungs receiving ONO-1301SR treatment, which did not differ significantly. These results suggest that prenatal ONO-1301SR treatment might have encouraged alveolar development in the nitrofen-induced CDH fetuses.

### Preservation of pulmonary arterial structure in CDH fetuses by ONO-1301SR treatment

**Fig 6:**
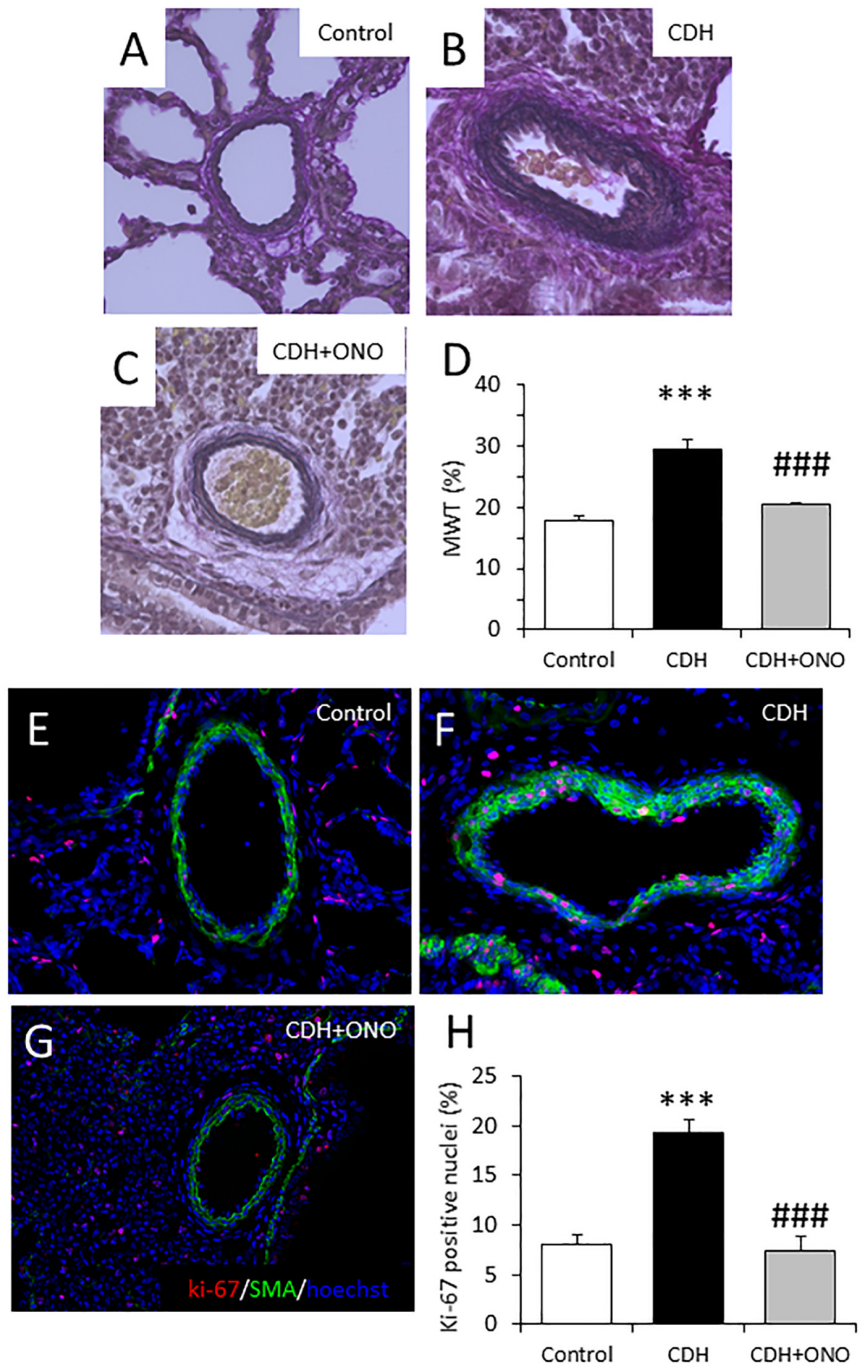
Preservation of pulmonary arterial structure in fetuses with congenital diaphragmatic hernia (CDH) following prenatal ONO-1301SR treatment. Representative van Gieson-stained lung sections from control (**A**), CDH (**B**), and CDH + ONO-1301SR-treated (**C**) fetuses at 400× magnification. The medial wall thickness (MWT, %) of control (white bar; n = 6), CDH (black bar; n = 6), and CDH + ONO-1301SR-treated (gray bar; n = 6) groups (**D**). Representative α-smooth muscle actin (α-SMA) and Ki-67 immunostaining in fetal lung sections from control (**E**), CDH (**F**), and CDH + ONO-1301SR-treated (**G**) pups at 400× magnification. The percentage of Ki-67-positive cells relative to the total number of cells within the medial layers of the pulmonary arteries in the lungs from control (white bar; n = 5), CDH (black bar; n = 6), and CDH + ONO-1301SR-treated (gray bar; n = 5) pups (**H**). The values are expressed as the mean ± SEM. *** *P* < 0.001 versus control fetuses; ### *P* < 0.001 versus nitrofen-CDH fetuses.

The structure of the pulmonary artery, which was considered to be the major target of the ONO-1301SR treatment, was assessed in the CDH fetuses relative to control fetuses by H&E staining and immunohistolabeling for α-SMA. The pulmonary arterial wall was markedly thickened with a dense accumulation of connective tissues around the artery in the CDH lungs without treatment relative to the controls and the CDH lungs receiving ONO-1301SR treatment, which showed preservation of pulmonary artery structure (Fig 6A–6C). The medial wall thickness in the CDH lungs without treatment was significantly higher than that in the other two groups, which showed comparable values (Fig 6D).

In addition, the proliferation activity of the pulmonary arterial smooth muscle cells in the CDH fetal lungs was assessed in comparison to the controls by immunohistolabeling for α-SMA and Ki-67. The α-SMA-positive layer surrounding the pulmonary artery was markedly thickened with a significantly higher number of Ki-67-positive cells in the CDH lungs without treatment than in the control lungs and those receiving ONO-1301SR treatment, which displayed a comparable number of α-SMA and Ki-67 double-positive cells (Fig 6E–6H).

**Fig 7:**
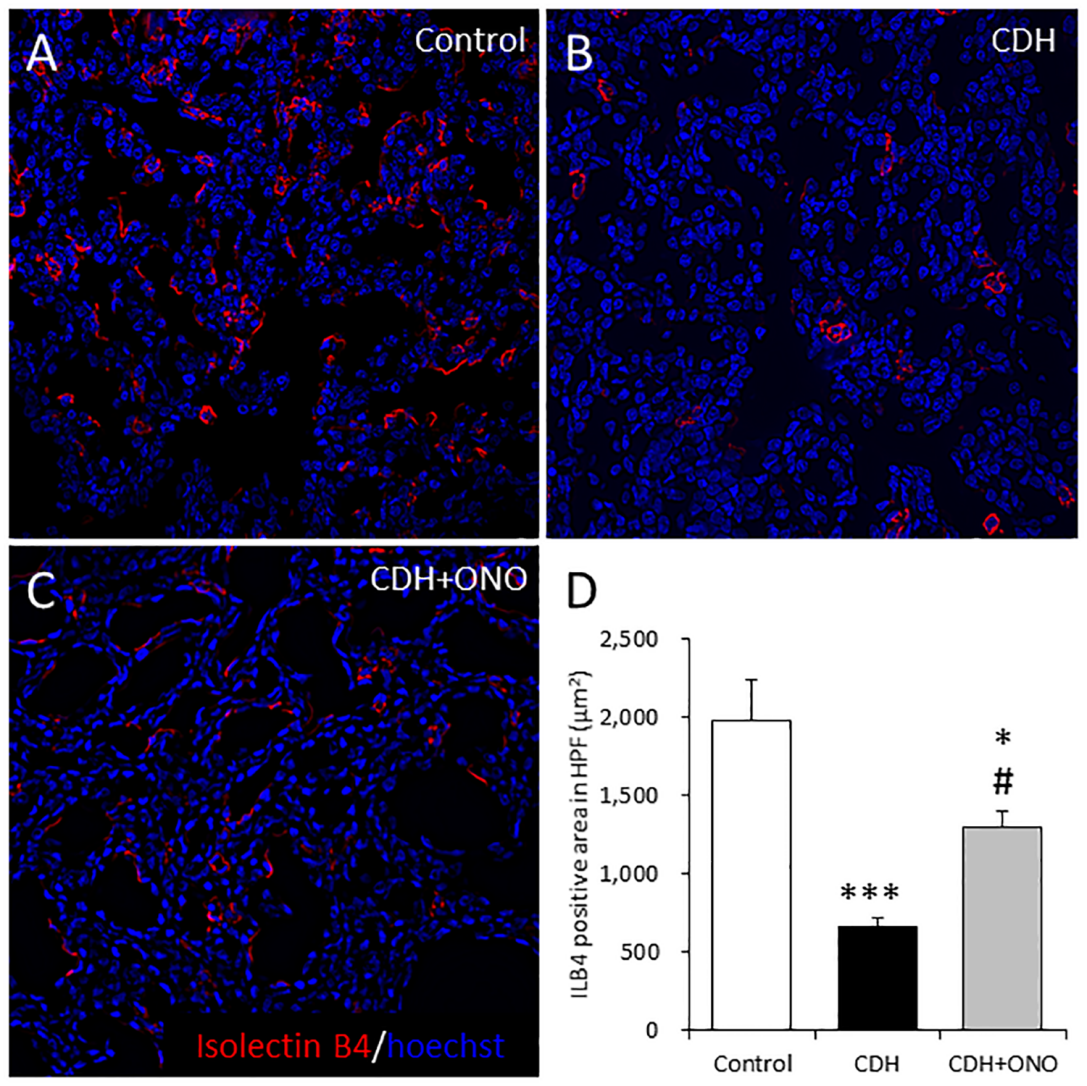
Enhanced pulmonary vascular bed development in fetuses with congenital diaphragmatic hernia (CDH) following prenatal ONO-1301SR treatment. Representative isolectin B4 (ILB4) staining in fetal lung sections from control (**A**), CDH (**B**), and CDH + ONO-1301SR-treated (**C**) pups at 400× magnification. The ILB4-positive area (μm^2^) in the lungs from control (white bar; n = 6), CDH (black bar; n = 6), and CDH + ONO-1301SR-treated (gray bar; n = 5) pups was calculated (**D**). The values are expressed as the mean ± SEM. * 0.01 < *P* < 0.05, *** *P* < 0.001 versus control fetuses; # 0.01 < *P* < 0.05 versus nitrofen-CDH fetuses.

Furthermore, the distribution and quantity of the pulmonary vascular bed in the CDH fetal lungs were assessed by ILB4 staining. The ILB4-positive pulmonary vascular bed was well distributed around the alveolar airspace in the controls, whereas the CDH lungs displayed a narrowed ILB4-positive pulmonary vascular bed (Fig 7A–7C). In addition, the CDH lungs, regardless of ONO-1301SR treatment, displayed a smaller ILB4-positive area than the controls, whereas the CDH lungs receiving ONO-1301SR treatment displayed a larger ILB4-positive area than those without treatment (Fig 7D).

### Upregulation of therapeutic factors in the CDH lung by prenatal ONO-1301SR treatment

**Fig 8:**
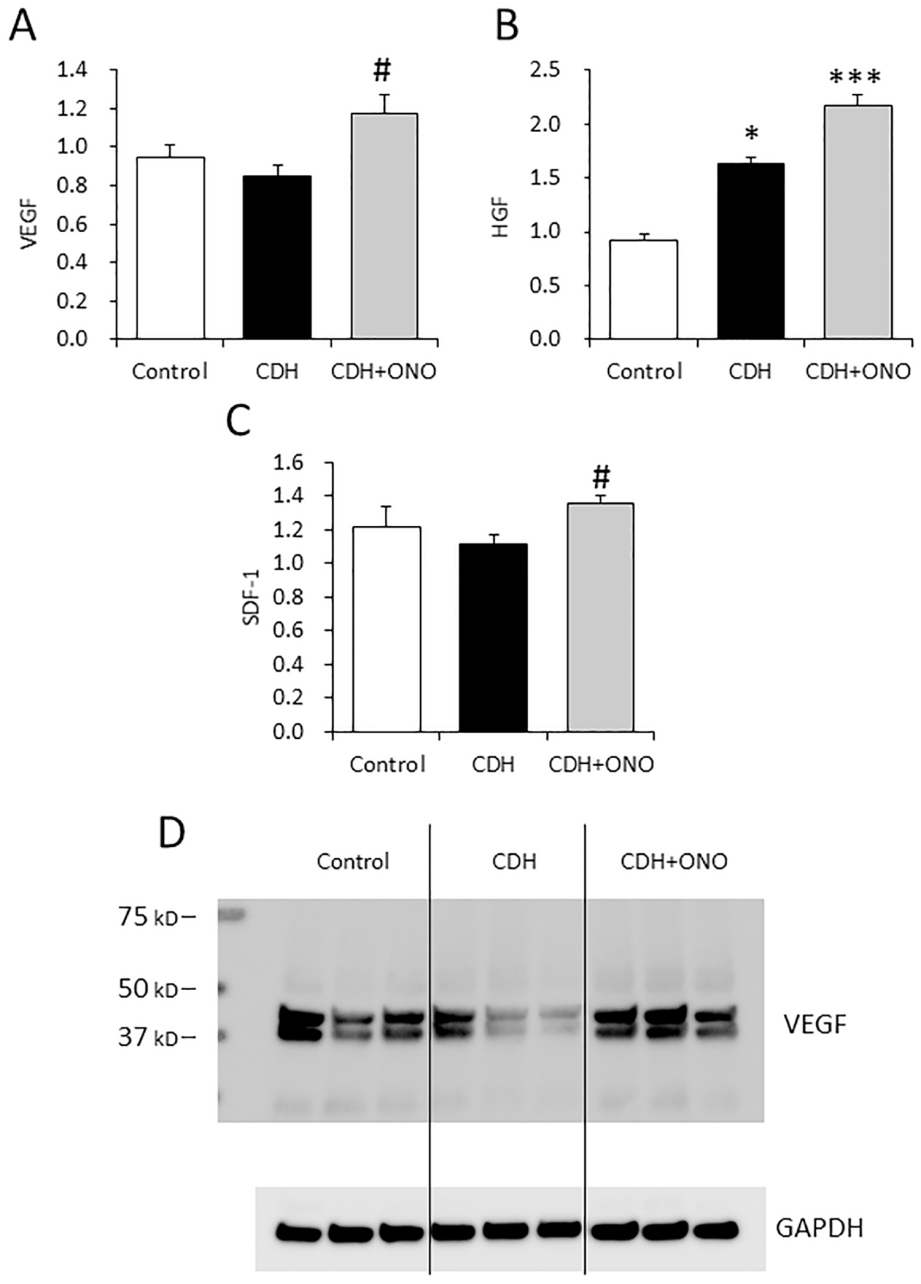
Upregulation of therapeutic factors in lungs with congenital diaphragmatic hernia (CDH) following prenatal ONO-1301SR treatment. Relative gene expression of *Vegf* (**A**), *Hgf* (**B**), and *Sdf1* (**C**) in the lungs from control (white bars; n = 5), CDH (black bars; n = 5), and CDH + ONO-1301SR-treated (gray bars; n = 5) pups. The average copy number of gene transcripts was normalized to that of the *Gapdh* gene. The values are expressed as the mean ± SEM. * 0.01 < *P*<0.05, *** *P* < 0.001 versus control fetuses; # 0.01 < *P* < 0.05 versus nitrofen-CDH fetuses. Representative immunoblots of VEGF protein levels in fetal lungs from the control, CDH, and CDH + ONO-130-treated pups (**D**).

The expression of VEGF, HGF, and SDF-1 in the CDH fetal lungs was assessed by quantitative RT-PCR and western blotting. The gene expression of *Vegf* and *Sdf1* did not differ between the CDH lungs without ONO-1301SR treatment and the control lungs, whereas *Hgf* expression was significantly higher in the untreated CDH lungs than in the controls. In contrast, the CDH lungs receiving ONO-1301SR treatment displayed significantly higher *Vegf*, *Hgf*, and *Sdf1* gene expression than the lungs without ONO-1301SR or the controls (Fig 8A–8C).

Western blotting revealed that VEGF protein expression in the CDH lungs without ONO-1301SR treatment was lower than that in the controls and that VEGF protein expression in CDH lungs receiving ONO-1301SR treatment was comparable to that of the controls (Fig 8D).

## Discussion

This study demonstrated that maternally administered ONO-1301SR entered the fetal blood through the placenta to enhance both alveolar and vascular development of the fetal lungs in the nitrofen-induced CDH rat model. Nitofen-induced CDH was successfully generated regardless of ONO-1301SR treatment. LW/BW was significantly higher in the ONO-1301SR-treated fetuses than in those without the treatment, in association with increased total DNA and protein contents in the fetal lungs. In addition, the pulmonary airspace and Ttf-1-positive alveolar epithelial cell numbers were significantly higher following ONO-1301SR treatment when compared to fetal lungs without the treatment. Furthermore, the medial wall thickness of the PA was preserved in the ONO-1301SR-treated group, and this effect was associated with fewer Ki-67-positive SMA double-positive cells in the PA wall relative to the no-treatment group. In addition, the ILB4-positive pulmonary vascular bed was significantly increased following ONO-1301SR treatment compared to the level without treatment. The expression of VEGF, HGF, and SDF-1 in the lungs was also significantly increased following ONO-1301SR treatment relative to the levels without treatment.

CDH is a congenital malformation causing severe respiratory distress with an incidence of approximately one per 2,000 live births [13], and it is associated with high mortality and morbidity rates [14]. The major pathology of this malformation is lung hypoplasia characterized by abnormalities in the alveolar/airway and vascular networks in the lung, and the degree of lung hypoplasia determines the clinical outcome of CDH [15]. Because CDH is prenatally diagnosed by ultrasonography in approximately 60% of cases [16], prenatal treatments for severe CDH have been advocated as theoretically ideal. Accordingly, a surgical approach by fetal endoscopic tracheal occlusion therapy, which is expected to enhance airway branching morphogenesis and pulmonary vasculature maturation, has recently been introduced as a prenatal treatment for severe CDH; however, the therapeutic efficacy of this treatment is reportedly inconsistent at least in part because of procedural variability [17, 18]. In addition, this treatment carries significant risks related to the surgical procedures, including preterm delivery. Thus, pharmacological approaches targeting lung development in the fetal period are being explored as non-invasive alternatives [12, 19, 20].

Hypoplastic lungs associated with CDH display pathologic abnormalities of major components such as the alveolar epithelium, airway, and vascular network. Notably, development of the alveolar epithelium and vascular network is known to be affected by expression of a variety of factors, including VEGF and HGF, in the prenatal period [21–25]. For example, infants with CDH present with a small airway space and low vessel number with abnormally muscularized PAs in association with reduced expression of angiogenic factors [26, 27]. The nitrofen-induced CDH rat model used in this study has been shown to pathologically and biologically mimic human lung hypoplasia associated with CDH, including the immature development of alveolar and vascular networks and muscularized PAs with reduced expression of angiogenic factors as further shown in this study. Direct and indirect supplementation of angiogenic factors to the fetus has therefore been examined using this model. In particular, prenatal maternal administration of sildenafil was reported to increase VEGF expression in the fetal lungs and attenuate the observed pulmonary vascular abnormalities [12], suggesting a possible “drug-repositioning” strategy of applying sildenafil to CDH. However, the outcomes of clinical studies have not yet been reported.

The present study explored the feasibility, safety, and therapeutic efficacy of maternal single injection of ONO-1301SR for treating CDH fetuses using the nitrofen-induced CDH rat model. In this study, ONO-1301 was shown to successfully enter the fetal blood, targeting its specific receptor, IPR, which is expressed in the vascular endothelium and smooth muscle cells in the fetal lung, to increase HGF, VEGF, and SDF-1 expression in the lung. The mechanisms underlying the effects of ONO-1301SR treatment are therefore considered to be chiefly angiogenic factor-related, as previously observed in the sildenafil study described above. The observed reduction of pulmonary arterial wall thickness in the CDH fetuses following treatment might represent a direct effect of the prostacyclin pathway [28] or indirect effects exerted through thromboxane A2 synthase inhibitory activity [29]. Effects of ONO-1301 on arterial wall thickness associated with pulmonary arterial hypertension consistent with the results of the present study have been previously reported [5].

Our results suggest that this treatment protocol might be suitable for clinical use. A clear advantage of ONO-1301SR treatment is that its effects are achieved by a single subcutaneous injection to the mother every 3 months. Previous reports exploring maternal interventions for treating fetal lung hypoplasia proposed frequently repeated or continuous administration of drugs such as sildenafil, imatinib, or bombesine. In addition, the dose and administration timing of ONO-1301SR were optimized prior to the present study. Several doses of ONO-1301SR (3, 10, or 30 mg/kg) and times for the treatment (E9.5, E11.5, or E13.5) were tested to enable selection of the most effective dose (30 mg/kg) and timing (E9.5) (data not shown).

In this study, administration of nitrofen and ONO-1301SR was conducted at E9.5, which corresponds to the period of organogenesis of rats. In addition, nitrofen induces various abnormalities of several organs such as the lung, diaphragm, heart, and kidney, which are also the therapeutic target organs of ONO-1301. Therefore, ONO-1301 might have a potential therapeutic effect for these organs as well. In this study, we did not examine the changes of the heart and kidney, and only focused on changes in the diaphragm and lung between groups treated with and without ONO-1301SR, which is indeed one of the limitations of this study. Moreover, there were no maternal complications associated with single injection of ONO-1301SR, such as death, infection, bleeding, behavioral alterations, or premature birth. Although proof of concept was established in this study, optimum timing of ONO-1301 SR administration and its dose are crucial tasks for clinical application of this treatment, needs to be investigated by several issues, such as diagnosis or maternal conditions taken into account. Further studies using large animal models or toxicity testing based on Good Laboratory Practice standards should be performed prior to launching clinical studies.

In conclusion, prenatal ONO-1301SR administration was protective against the progression of lung hypoplasia associated with CDH in the nitrofen-induced rat model, indicating the potential of this treatment for pathologies exhibiting lung hypoplasia.

## References

[1] Taira Y, Miyazaki E, Ohshiro K, Yamataka T, Puri P. Administration of antenatal glucocorticoids prevents pulmonary artery structural changes in nitrofen-induced congenital diaphragmatic hernia in rats. J Pediatr Surg. 1998;33: 1052–1056. 9694093 10.1016/s0022-3468(98)90530-9

[2] Lemus-Varela Mde L, Soliz A, Gómez-Meda BC, Zamora-Perez AL, Ornelas-Aguirre JM, Melnikov V, et al Antenatal use of bosentan and/or sildenafil attenuates pulmonary features in rats with congenital diaphragmatic hernia. World J Pediatr. 2014;10: 354–359. 10.1007/s12519-014-0512-y 25515807

[3] Schmidt AF, Gonçalves FL, Regis AC, Gallindo RM, Sbragia L. Prenatal retinoic acid improves lung vascularization and VEGF expression in CDH rat. Am J Obstet Gynecol. 2012;207: 76.e25–32. 10.1016/j.ajog.2012.04.025 22621815

[4] Imanishi Y, Miyagawa S, Fukushima S, Ishimaru K, Sougawa N, Saito A, et al Sustained-release delivery of prostacyclin analogue enhances bone marrow-cell recruitment and yields functional benefits for acute myocardial infarction in mice. PLoS One. 2013;8: e69302 10.1371/journal.pone.0069302 23894446 PMC3716598

[5] Kataoka M, Nagaya N, Satoh T, Itoh T, Murakami S, Iwase T, et al A long-acting prostacyclin agonist with thromboxane inhibitory activity for pulmonary hypertension. Am J Respir Crit Care Med. 2005;172: 1575–1580. 16192456 10.1164/rccm.200501-102OC

[6] Ishimaru K, Miyagawa S, Fukushima S, Saito A, Sakai Y, Ueno T, et al Synthetic prostacyclin agonist, ONO1301, enhances endogenous myocardial repair in a hamster model of dilated cardiomyopathy: a promising regenerative therapy for the failing heart. J Thorac Cardiovasc Surg. 2013;146: 1516–1525. 10.1016/j.jtcvs.2013.02.045 24229503

[7] Obata H, Sakai Y, Ohnishi S, Takeshita S, Mori H, Kodama M, et al Single injection of a sustained-release prostacyclin analog improves pulmonary hypertension in rats. Am J Respir Crit Care Med. 2008;177: 195–201. 17975203 10.1164/rccm.200703-349OC

[8] Okoye BO, Losty PD, Lloyd DA, Gosney JR. Effect of prenatal glucocorticoids on pulmonary vascular muscularisation in nitrofen-induced congenital diaphragmatic hernia. J Pediatr Surg. 1998;33: 76–80. 9473105 10.1016/s0022-3468(98)90366-9

[9] Kamiyama M, Usui N, Kamata S, Fukuzawa M, Nagaya N, Kangawa K. Adrenomedullin is up-regulated in nitrofen-induced fetal pulmonary hypoplasia. J Pediatr Surg. 2005;40: 1562–1567. 16226985 10.1016/j.jpedsurg.2005.06.005

[10] Thurlbeck WM. Internal surface area and other measurements in emphysema. Thorax. 1967;22: 483–496. 5624577 10.1136/thx.22.6.483 PMC471691

[11] Kanai M, Kitano Y, von Allmen D, Davies P, Adzick NS, Flake AW. Fetal tracheal occlusion in the rat model of nitrofen-induced congenital diaphragmatic hernia: Tracheal occlusion reverses the arterial structural abnormality. J Pediatr Surg. 2001;36: 839–845. 11381408 10.1053/jpsu.2001.23950

[12] Luong C, Rey-Perra J, Vadivel A, Gilmour G, Sauve Y, Koonen D, et al Antenatal sildenafil treatment attenuates pulmonary hypertension in experimental congenital diaphragmatic hernia. Circulation. 2011;123: 2020–2131. 21537000 10.1161/CIRCULATIONAHA.108.845909

[13] Langham MR Jr, Kays DW, Ledbetter DJ, Frientzen B, Sanford LL, Richards DS. Congenital diaphragmatic hernia. Epidemiology and outcome. Clin Perinatol. 1996;23: 671–688. 8982563

[14] Leeuwen L, Fitzgerald DA. Congenital diaphragmatic hernia. J Paediatr Child Health. 2014;50: 667–673. 10.1111/jpc.12508 24528549

[15] Thébaud B, Mercier JC, Dinh-Xuan AT. Congenital diaphragmatic hernia. A cause of persistent pulmonary hypertension of the newborn which lacks an effective therapy. Bio Neonate. 1998;74: 323–336. 9742261 10.1159/000014050

[16] Garne E, Haeusler M, Barisic I Gjergja Stoll C, Clementi M; Euroscan Study Group. Congenital diaphragmatic hernia: evaluation of prenatal diagnosis in 20 European regions. Ultrasound Obstet Gynecol. 2002;19: 329–333. 11952959 10.1046/j.1469-0705.2002.00635.x

[17] Jani JC, Nicolaides KH, Gratacós E, Valencia CM, Doné E, Martinez JM, et al Severe diaphragmatic hernia treated by fetal endoscopic tracheal occlusion. Ultrasound Obstet Gynecol. 2009;34: 304–310. 10.1002/uog.6450 19658113

[18] Ruano R, Yoshisaki CT, da Silva MM, Ceccon ME, Grasi MS, Tannuri U, et al A randomized controlled trial of fetal endoscopic tracheal occlusion versus postnatal management of severe isolated congenital diaphragmatic hernia. Ultrasound Obstet Gynecol. 2012;39: 20–27. 10.1002/uog.10142 22170862

[19] Chang YT, Ringman Uggla A, Osterholm C, Tran PK, Eklӧf AC, Lengquist M, et al Antenatal imatinib treatment reduces pulmonary vascular remodeling in a rat model of congenital diaphragmatic hernia. Am J Physiol Lung Cell Mol Physiol. 2012; 302: L1159–L1166. 10.1152/ajplung.00325.2010 22447953

[20] Sakai K, Kimura O, Furukawa T, Fumino S, Higuchi K, Wakao J, et al Prenatal administration of neuropeptide bombesin promotes lung development in a rat model of nitrofen-induced congenital diaphragmatic hernia. J Pediatr Surg. 2014;49: 1749–1752. 10.1016/j.jpedsurg.2014.09.015 25487476

[21] Jakkula M, Le Cras TD, Gebb S, Hirth KP, Tuder RM, Voelkel NF, et al Inhibition of angiogenesis decreases alveolarization in the developing rat lung. Am J Physiol Lung Cell Mol Physiol. 2000;279: L600–L607. 10956636 10.1152/ajplung.2000.279.3.L600

[22] Kunig AM, Balasubramaniam V, Markham NE, Morgan D, Montgomery G, Grover TR, et al Recombinant human VEGF treatment enhances alveolarization after hyperoxic lung injury in neonatal rats. Am J Physiol Lung Cell Mol Physiol. 2005;289: L529–L535. 15908474 10.1152/ajplung.00336.2004

[23] Kunig AM, Balasubramaniam V, Markham NE, Seedorf G, Gien J, Abman SH. Recombinant human VEGF treatment transiently increases lung edema but enhances lung structure after neonatal hyperoxia. Am J Physiol Lung Cell Mol Physiol. 2006;291: L1068–L1078. 16829629 10.1152/ajplung.00093.2006

[24] Thébaud B, Abman SH. Bronchopulmonary dysplasia: where have all the vessels gone? Roles of angiogenic growth factors in chronic lung disease. Am J Respir Crit Care Med. 2007;175: 978–985. 17272782 10.1164/rccm.200611-1660PP PMC2176086

[25] Thébaud B, Ladha F, Michelakis ED, Sawicka M, Thurston G, Eaton F, et al Vascular endothelial growth factor gene therapy increases survival, promotes lung angiogenesis, and prevents alveolar damage in hypoxia-induced lung injury: evidence that angiogenesis participates in alveolarization. Circulation. 2005;112: 2477–2486. 16230500 10.1161/CIRCULATIONAHA.105.541524

[26] Hislop A, Reid L. Persistent hypoplasia of the lung after repair of congenital diaphragmatic hernia. Thorax. 1976;31: 450–455. 968803 10.1136/thx.31.4.450 PMC470458

[27] Yamataka T, Puri P. Pulmonary artery structural changes in pulmonary hypertension complicating congenital diaphragmatic hernia. J Pediatr Surg. 1997;32: 387–390. 9093999 10.1016/s0022-3468(97)90587-x

[28] Wharton J, Davie N, Upton PD, Yacoub MH, Polak JM, Morrell NW. Prostacyclin analogues differentially inhibit growth of distal and proximal human pulmonary artery smooth muscle cells. Circulation. 2000;102: 3130–3136. 11120706 10.1161/01.cir.102.25.3130

[29] Budhiraja R, Tuder RM, Hassoun PM. Endothelial dysfunction in pulmonary hypertension. Circulation. 2004;109: 159–165. 14734504 10.1161/01.CIR.0000102381.57477.50

